# 手术在非小细胞肺癌单发脑转移治疗中的作用分析

**DOI:** 10.3779/j.issn.1009-3419.2013.12.05

**Published:** 2013-12-20

**Authors:** 皓 白, 宝惠 韩

**Affiliations:** 200030 上海，上海交通大学医学院附属上海市胸科医院呼吸内科 Department of Respiratory Medicine, Shanghai Chest Hospital, School of Medicine, Shanghai Jiaotong University, Shanghai 20030, China

**Keywords:** 肺肿瘤, 脑转移, 手术切除, Lung neoplasms, Brain metastases, Surgical resection

## Abstract

**背景与目的:**

非小细胞肺癌脑转移较多见，常用的治疗方法有放疗、化疗等，但预后不佳。本研究拟了解手术在非小细胞肺癌同步单发脑转移综合治疗中的作用。

**方法:**

回顾性分析46例非小细胞肺癌同步单发脑转移患者的临床资料。所有患者接受肺部肿瘤切除，术后行全脑放疗及化疗。其中13例患者行脑转移瘤切除，33例患者行脑部立体定向放射治疗。

**结果:**

本组患者总的中位生存期为16.8个月，1年、2年和3年生存率分别为76.1%、20.9%和4.7%。脑转移瘤手术组和立体定向治疗组的中位生存期分别为18.3个月和15. 8个月（*P*=0.091, 2）。

**结论:**

手术切除肺部原发肿瘤及脑转移瘤对改善非小细胞肺癌单发脑转移患者的预后具有一定作用。脑转移瘤手术切除或立体定向治疗对患者的生存期未见有影响。

## Abstract (en)

**Background and objective:**

Brain metastases are common in non-small cell lung cancer. Usual treatments include radiotherapy and chemotherapy. However, these methods result in poor patient prognosis. The aim of this study is to assess the effectiveness of surgical resection in the multimodality management of non-small cell lung cancer patients with synchronous solitary brain metastasis.

**Methods:**

The clinical data of 46 non-small cell lung cancer patients with synchronous solitary brain metastasis were retrospectively reviewed. All patients underwent surgical resection of primary lung tumor, followed by whole brain radiotherapy and chemotherapy. In addition, 13 out of the 46 patients underwent resection of brain metastasis, whereas the remaining 33 patients received stereotactic radiosurgery.

**Results:**

The median survival time of the enrolled patients was 16.8 months. The 1-, 2-, and 3-year survival rates were 76.1%, 20.9%, and 4.7%, respectively. The median survival times of the patients with brain metastasis resection or stereotactic radiosurgery were 18.3 and 15.8 months, respectively (*P*=0.091, 2).

**Conclusion:**

Surgical resection of primary lung tumor and brain metastasis may improve prognosis of non-small cell lung cancer patients with synchronous solitary brain metastasis. However, the survival benefit of surgical resection over brain metastasis resection or stereotactic radiosurgery is uncertain.

肺癌是最常见的恶性肿瘤之一，非小细胞肺癌（non-small cell lung cancer, NSCLC）约占85%，5年生存率仅16%^[1, 2]^。NSCLC脑转移的发生率约为30%-50%^[3]^，常见的治疗方法有全脑放疗（whole brain radiotherapy, WBRT）、立体定向放射治疗（stereotaetic radiosurgery, SRS）及化疗等。对于肺部病灶可切除的NSCLC单发脑转移患者，既往认为临床属于IV期，故一般仅给予化疗或放疗等治疗。近年来随着诊断和治疗水平的提高，采取手术方法选择性切除肺部原发病灶及脑转移瘤的临床意义受到关注，但目前对于此类患者进行手术治疗的可行性及有效性仍有争议。本研究拟回顾性分析46例NSCLC单发脑转移患者的临床资料，对手术在NSCLC单发脑转移综合治疗中的作用进行探讨。

## 1 资料与方法

### 1.1 一般情况

收集2001年-2011年上海交通大学附属胸科医院诊治的46例NSCLC脑转移患者的临床资料（均具有病理学依据）。脑转移根据脑部增强CT或核磁共振成像（magnetic resonance imaging, MRI）确定，均为单发转移灶（最大直径0.8 cm-4.2 cm）；本文所指的脑转移是指NSCLC诊断同时发现有单发脑转移并且没有颅外其它脏器的转移，不包括NSCLC治疗后继发出现的脑转移。患者中位年龄51岁（37岁-66岁）；男性34例，女性12例；体能状态（performance status, PS）评分0-1分39例，2分7例；腺癌33例，鳞癌6例，腺鳞癌4例，大细胞癌3例；周围型37例，中央型9例；肺癌术后T分期：T1 12例，T2 25例，T3 9例；区域淋巴结（N）: N0 7例，N1 26例，N2 13例。脑转移部位：大脑38例，小脑8例；有脑转移症状35例，无脑转移症状11例（表 1）。

**1:** 患者的临床特征 Clinical characteristics of the patients

Clinical characteristic		*n* (%)
Age (yr)	< 60	36 (78.3%)
	≥60	10 (21.7%)
Gender	Male	34 (73.9%)
	Female	12 (26.1%)
PS score	0-1	39 (84.8%）
	2	7 (15.2%)
Histology	Adenocarcinoma	33 (71.8%)
	Squamous cell carcinoma	6 (13.0%)
	Ad-sq carcinoma	4 (8.7%)
	Large cell carcinoma	3 (6.5%)
Location of lung cancer	Peripheral type	37 (80.4%)
	Central type	9 (19.6%)
T stage	T1	12 (26.1%)
	T2	25 (54.3%)
	T3	9 (19.6%)
Regional lymph node	N0	7 (15.2%)
	N1	26 (56.5%)
	N2	13 (28.3%)
Site of brain metastases	Cerebrum	38 (82.6%)
	Cerebellum	8 (17.4%)
Metastatic symptoms	Yes	35 (76.1%)
	No	11 (23.9%)

### 1.2 治疗方法

① 所有患者行肺部肿瘤手术切除（肺叶切除41例，全肺切除5例）。②脑转移瘤手术切除13例（肺部手术前3周-4周），SRS治疗33例（肺部手术前24例，肺部手术后9例）。③所有患者适时进行WBRT，放射源为6 MV或8 MV的*χ*射线，采用全脑两侧野对穿照射，放射剂量为30 Gy/10次/2周。④所有患者接受含铂方案的化疗，药物包括鬼臼噻吩甙、环己亚硝脲、长春瑞宾、吉西他滨、紫杉醇、多西他赛、培美曲塞等，中位化疗疗程5个周期（2-10周期）。⑤3例患者在放化疗后口服靶向药物治疗（易瑞沙2例，特罗凯1例）。

### 1.3 分析指标

生存期和生存率从脑转移诊断日起计算。

### 1.4 统计学方法

采用SPSS 11.0软件中的*Kaplan Meier*法进行生存分析并进行*Log-rank*检验组间差异，描绘生存曲线并分析比较生存期和生存率，*P* < 0.05为差异有统计学意义。死亡病例为截尾数据，存活病例为未截尾数据。

## 2 结果

### 2.1 患者随访

本组患者随访截止日期为2013年6月30日，其中死亡45例，存活1例。中位生存时间（median survival time, MST）为16.8个月（6.9个月-38.2个月），1年、2年和3年生存率分别为76.1%、20.9%和4.7%（图 1）。单因素分析显示生存期与患者的年龄、性别、PS评分、病理类型、肺部肿瘤的部位、肺癌术后T分期、N分期等均未见有相关性（*P*>0.05）。

**1:**
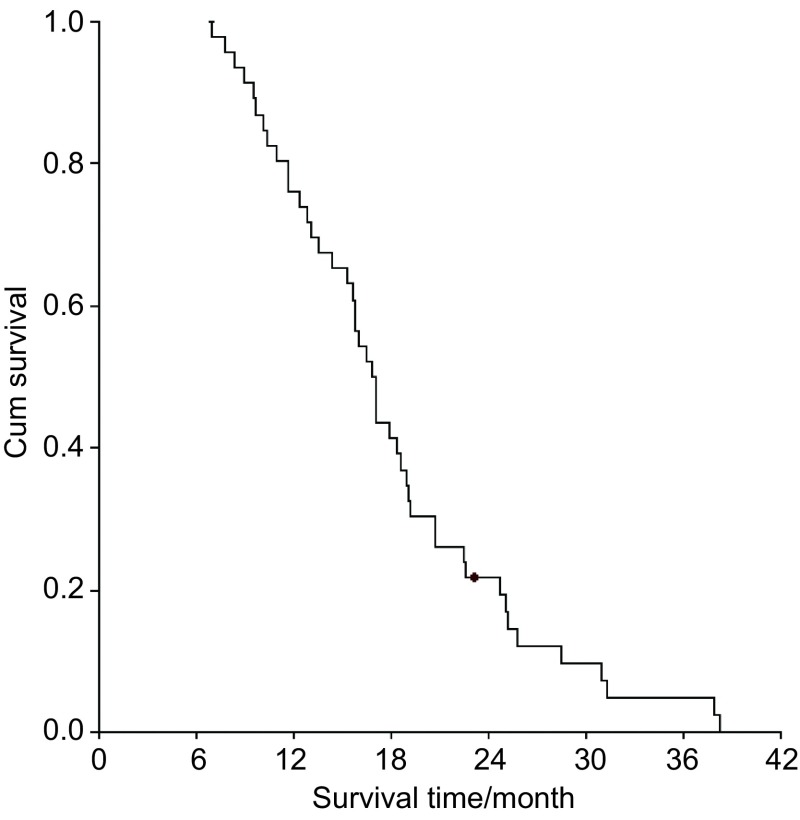
患者的生存曲线 Survival curve of the patients

### 2.2 生存情况

脑转移瘤切除和SRS患者的MST分别为18.3个月和15.8个月，1年、2年、3年生存率分别为84.6%、36.3%、9.1%和72.7%、15.1%、3.0%（*P*=0.091, 2）（图 2）。

**2:**
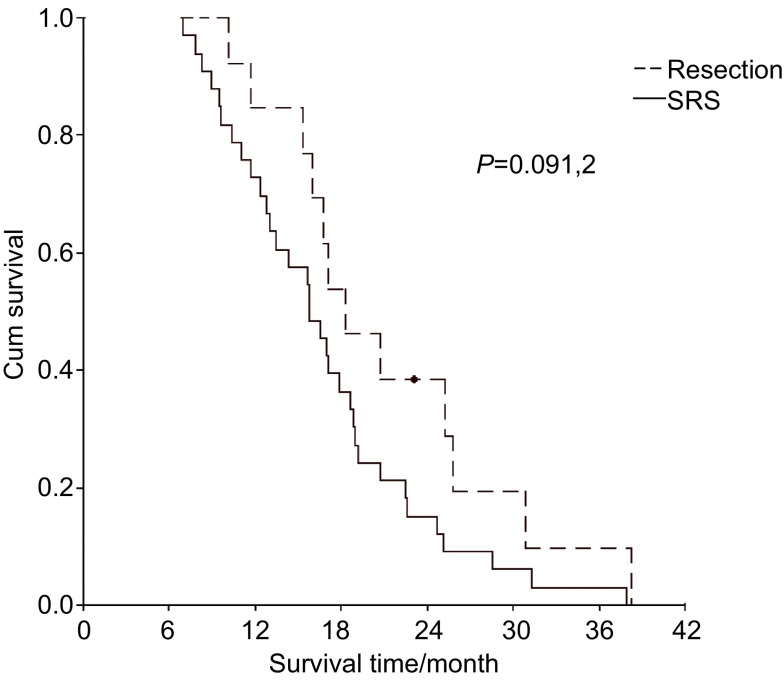
脑转移瘤切除和立体定向治疗的患者的生存曲线 Survival curve of patients with brain metastasectomy *vs* SRS. SRS: stereotaetic radiosurgery.

## 3 讨论

NSCLC脑转移较为常见，其中约1/3的患者确诊时仅表现为脑部孤立性病灶而没有其它脏器转移^[4]^，这为手术提供了可能。早期认为此类患者在切除肺部原发肿瘤后并不具有生存获益，因此对肺部病灶不需要积极外科治疗。近年来发现伴孤立性单发脑转移的NSCLC患者，选择性切除肺部病灶可以改善预后，其MST为20.5个月-64.9个月^[5-8]^，5年生存率约21%-36%^[6-8]^。本组46例患者均进行了肺部原发肿瘤切除术，术后的MST为16.8个月，但3年生存率较低且无存活超过5年的病例，与相关文献的数据有所不同。

文献^[8-12]^报道称NSCLC脑转移患者肺部手术的预后与纵隔淋巴结的分期有关，N0患者的预后最好，N1患者的预后则优于N2患者。但也有报道患者的预后与纵隔淋巴结分期无关^[6]^或与肺部病灶的T分期有关^[7]^。本组资料显示患者肺部手术的预后与纵隔淋巴结分期或肺部病灶的T分期均不相关，与有些学者^[6, 13]^的报道相近。目前一般认为同期单发脑转移的NSCLC肺部原发病灶的治疗，N0-1的患者可以考虑肺部手术，而N2-3的患者以放化疗为主^[3, 9]^。美国国立综合癌症网络（National Comprehensive Cancer Network, NCCN）2013年临床指南则建议初诊确定有孤立性脑转移的NSCLC患者，对于N0和T1-T3N1的肺部病灶可选择性考虑手术切除。本研究显示对有适应症的患者切除肺部原发肿瘤，能够延长其生存时间，但是否具有长期生存获益则尚不肯定，仍有待于进一步探讨。由于本组资料中包括了一定比例的N2的患者，并且患者在肺部手术同时还接受了脑转移瘤切除或SRS、放疗及化疗等多种治疗，对总体预后的判断可能会造成一定影响。此外，肺部原发肿瘤是否完全切除与术后的复发及预后有关^[8, 13]^。我们认为尽管NSCLC伴脑转移属于晚期，但在肺部手术时仍应遵循彻底根治的原则，最大限度切除肿瘤组织及常规清扫区域淋巴结，力求最佳的治疗效果。

近年来随着显微神经外科技术的提高，手术切除单发脑转移瘤逐渐为人们所接受。手术可以解除肿瘤对脑组织压迫、降低颅内压、改善神经机能状态及为后续治疗创造条件，因此对单发脑转移患者可以选择性地手术切除脑转移瘤^[14, 15]^。通常认为NSCLC脑转移患者手术切除脑转移瘤的适应症是：①全身情况较好、无恶液质或重要器官功能不全并且可以耐受手术；②肺部原发病灶能够切除；③单发或相邻部位两个孤立性脑转移灶，肿瘤最大直径 < 5 cm；④无颅外其它部位转移^[16-18]^。文献^[19-21]^报道NSCLC患者脑转移瘤手术切除后的MST为7.8个月-13.2个月，显示出一定的生存获益。但近年来也有研究表明对手术可切除的脑转移瘤患者，采取SRS同样可以取得较好的治疗效果，远期生存接近手术^[9, 5, 22, 23]^。本组资料中脑转移瘤切除患者的MST和生存率稍好于SRS的患者，但未见有统计学差异。由于本研究为回顾性资料，患者的预后还受其它到因素的影响，如PS评分、肺部原发灶的分期、脑转移瘤大小、WBRT敏感性及不同化疗方案的疗效等，因此现有数据尚不足以判断脑转移瘤手术切除和SRS的优劣，如能积累更多的病例将有助于获取更有价值的信息。但笔者以为手术切除脑转移瘤在NSCLC单发脑转移的多学科综合治疗中的作用不应被忽视，而SRS具有快速、安全、定位精确的优势，为无法耐受脑部手术的患者提供了又一种治疗选择。

关于肺部原发肿瘤和脑部转移瘤同时手术切除的文献较少。有报道NSCLC脑转移患者联合手术后的MST为11个月-23个月^[24-27]^，1年、2年和3年生存率分别为56%-95%、27.3%-47%和9.0%-14%^[25-27]^，这些研究结果各有所不同，可能与病例的多少、来源及不同的评价方法有关。本资料中有13例患者进行了肺部肿瘤和脑转移瘤联合手术切除，MST为19.5个月，1年、2年和3年生存率分别为84.6%、36.2%和9.1%，且未出现术后死亡的情况，初步表明部分患者可以从联合手术中获益，同时在临床实践中把握手术指征尤为重要。笔者以为对有适应症的NSCLC脑转移患者，可慎重采取联合手术的治疗策略。

WBRT有助于消除脑部潜在的微转移灶、减少复发，提高无脑转移灶的生存时间^[28-30]^，而常规的脑CT或MRI通常不能发现这些微转移灶。此外，脑转移表明患者体内存在癌细胞的血道转移，因此有必要对NSCLC脑转移患者进行化疗^[30, 31]^。本组病例在手术后均接受了WBRT及含铂类药物的化疗，取得了较好的疗效。笔者认为WBRT及化疗在NSCLC脑转移综合治疗中具有不可或缺的重要作用。

NSCLC脑转移的治疗因临床症状的多样性、复杂性及患者的个体差异而缺乏成熟的共识。虽然国内外颁布了相关治疗指南，但学术界仍有不同观点。本研究表明在严格掌握适应症的前提下，手术切除肺部原发肿瘤及脑转移瘤对改善NSCLC单发脑转移患者的预后具有一定作用，术后采取放疗、化疗及靶向药物等多学科综合治疗，有助于进一步提高其生活质量及延长生存期。由于本研究属于回顾性临床观察，病例数较少且存在着高度选择性，具有一定的局限性，如能进行大样本随机对照研究会更有说服力。
